# A mitochondria-targeted chemiluminescent probe for detection of hydrogen sulfide in cancer cells, human serum and *in vivo*†

**DOI:** 10.1039/d3cb00070b

**Published:** 2023-07-18

**Authors:** Hande Gunduz, Toghrul Almammadov, Musa Dirak, Alperen Acari, Berkan Bozkurt, Safacan Kolemen

**Affiliations:** a Nanofabrication and Nanocharacterization Center for Scientific and Technological Advanced Research, Koç University Istanbul 34450 Turkey; b Department of Chemistry, Koç University, Rumelifeneri Yolu Istanbul 34450 Turkey skolemen@ku.edu.tr; c Koç University Research Center for Translational Medicine (KUTTAM) Istanbul 34450 Turkey; d Graduate School of Health Sciences, Koç University, Rumelifeneri Yolu Istanbul 34450 Turkey; e Koç University Surface Science and Technology Center (KUYTAM) Istanbul 34450 Turkey

## Abstract

Hydrogen sulfide (H_2_S) as a critical messenger molecule plays vital roles in regular cell function. However, abnormal levels of H_2_S, especially mitochondrial H_2_S, are directly correlated with the formation of pathological states including neurodegenerative diseases, cardiovascular disorders, and cancer. Thus, monitoring fluxes of mitochondrial H_2_S concentrations both *in vitro* and *in vivo* with high selectivity and sensitivity is crucial. In this direction, herein we developed the first ever example of a mitochondria-targeted and H_2_S-responsive new generation 1,2-dioxetane-based chemiluminescent probe (MCH). Chemiluminescent probes offer unique advantages compared to conventional fluorophores as they do not require external light irradiation to emit light. MCH exhibited a dramatic turn-on response in its luminescence signal upon reacting with H_2_S with high selectivity. It was used to detect H_2_S activity in different biological systems ranging from cancerous cells to human serum and tumor-bearing mice. We anticipate that MCH will pave the way for development of new organelle-targeted chemiluminescence agents towards imaging of different analytes in various biological models.

## Abstract 2

Mitochondria-targeted chemiluminescent probe MCH enables visualization of hydrogen sulfide activity in cancer cells, human serum, and animal models.

## Graphical abstract

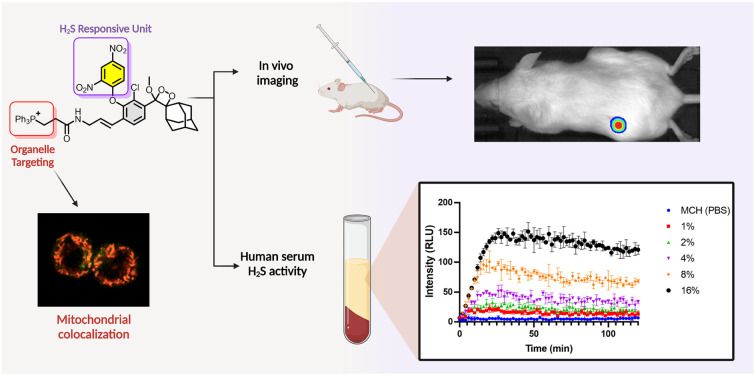

## Introduction

Hydrogen sulfide (H_2_S) is a well-known gasotransmitter, and is mainly synthesized by enzymatic pathways.^1–4^ Cystathionine β-synthase (CBS), cystathionine γ-lyase (CSE), and 3-mercaptopyruvate sulfur transferase (MPST) are three major enzymes responsible for the synthesis of H_2_S in mammalian cells, in which cysteine is the major substrate.^5–7^ It is produced in the central nervous system, blood, gastrointestinal tract, and heart.^5,8–14^ H_2_S is a critical antioxidant molecule that contributes to reactive oxygen species (ROS) mediated signaling and redox homeostasis.^15–18^ Thus, it serves as an effective cytoprotective agent as it can effectively neutralize numerous reactive oxygen species.^19–23^ Additionally, it plays a central role in cell proliferation, regulation of inflammation, insulin release, angiogenesis, migration, and apoptosis.^24–35^ On the other side, abnormal levels of H_2_S lead to cell malfunction and are directly associated with several pathological states including Alzheimer's diseases, cancer, cardiovascular disorders, hypertension, diabetes, depression and Down syndrome.^36–40^ The H_2_S level in the blood plasma is around 10–100 μM and fluctuations in the serum H_2_S concentration can be utilized as an indication of a disease formation.^13^

CBS and CSE are known to function mainly in the cytosol.^41^ However, under hypoxic conditions, which are very likely to be seen in many solid tumors, CSE translocates to mitochondria and catalyzes the conversion of cysteine to H_2_S.^42^ H_2_S can also be generated with the consecutive action of aspartate aminotransferase (AAT) and 3-mercaptopyruvate sulfur transferase (MPST) in the mitochondria.^5^ Mitochondrial H_2_S generation has been shown to regulate energy metabolism by increasing ATP production, which supports cell survival under hypoxia.^43–45^ Accordingly, it contributes to hypoxia resistance of cancer cells. Furthermore, it has been reported that H_2_S downregulates the ROS production in mitochondria and thereby protects mitochondria from oxidative stress.^46,47^ Dysregulation of mitochondrial H_2_S is also linked to the formation of previously mentioned diseases.^48,49^ Considering its role in physiological and pathological processes and its unique activity in mitochondria, it is of great importance to monitor mitochondrial H_2_S fluxes, specifically in cancer cells, in both cell cultures and *in vivo* with high selectivity and sensitivity. Additionally, tools that can detect H_2_S levels in human serum are highly critical in pathology.

Traditional approaches for H_2_S detection involve colorimetric and electrochemical assays along with chromatographic techniques (gas and ion-exchange chromatography, HPLC), in which complicated instruments and sample preparation steps are needed.^50–57^ Furthermore, these methods cause damage to cells and cannot offer precise detection. To address the drawbacks of conventional techniques, fluorescent probes that can offer real-time imaging opportunities, high spatial and temporal resolution, and selectivity without destruction of cells or tissues are highly promising. To this end, numerous activity-based fluorophores have been introduced to image endogenous H_2_S activity^58–61^ including several probes targeting mitochondria.^62–65^ H_2_S has also been used for prodrug activation in numerous literature examples owing to its correlation with tumorogenesis.^5,45,48^ Common H_2_S-triggered reactions that have been used in the activation of these agents and probes are based on nucleophilic addition, thiolysis, reduction of the azide and copper precipitation.^61,66–68^ Although highly valuable results have been obtained with conventional fluorophores, their intrinsic problems such as the need for external light irradiation, limited penetration of the excitation light, and the autofluorescence-induced low signal-to-noise ratio limit their broader acceptance and application areas, especially in *in vivo* models.

New generation phenoxy 1,2-dioxetane-based chemiluminescent probes bearing electron-withdrawing acrylate units have attracted great attention in recent years as an alternative to fluorescent probes.^69–77^ Chemiluminescent probes do not require external light irradiation to emit light and offer a very high signal-to-noise ratio along with a high selectivity and sensitivity.^78–80^ Additionally, they bear a modular scaffold, which can be simply modified toward the development of activity-based probes. Accordingly, new generation 1,2-dioxetane derivatives have been developed to monitor numerous analytes ranging from enzymes to ROS and biothiols including H_2_S.^81–86^ Very recently, we have introduced the first ever example of a mitochondria-targeted chemiluminescence agent by modifying the phenoxy 1,2-dioxetane core, which was responsive to the leucine aminopeptidase (LAP) enzyme, with a well-established mitochondrion targeting triphenylphosphonium (TPP) group.^87^ Cationic nature of TPP is critical to ensure the attraction between the probe and the negatively charged membrane of mitochondria as previously shown in the literature.^87^ We showed that TPP modification does not affect the luminescence characteristics of dioxetanes, and the probe can be used for *in vivo* imaging.

**Fig. 1:**
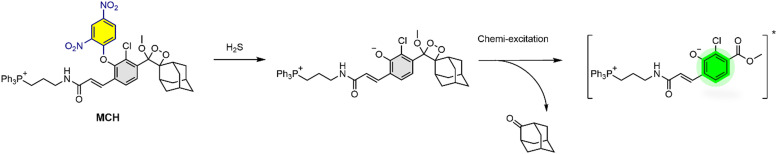
Structure of MCH and its activation with H_2_S.

In this study, we turned our attention to H_2_S visualization and developed a H_2_S-activatable and mitochondria-targeted 1,2-dioxetane-based chemiluminescence agent (MCH) (Fig. 1) for the first time to monitor H_2_S activity in cell cultures, human serum and *in vivo*. MCH was constructed on our TPP bearing adamantyl-phenoxy 1,2-dioxetane core and linked to a H_2_S-responsive dinitrophenyl group^88,89^ through an ether linkage as a masking unit. When the phenol was masked, MCH showed no luminescence signal. H_2_S-mediated nucleophilic aromatic substitution cleaved the dinitrophenyl group and the phenolate was generated, which was followed by the chemiexcitation process to yield a strong luminescence signal (Fig. 1).

## Results and discussion

### Synthesis

**Scheme 1:**
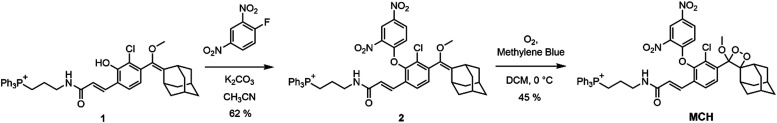
Synthesis of MCH.

The synthesis of MCH is depicted in Scheme 1. TPP-bearing core (1) was synthesized by following our previous study.^87^ Then, (1) was reacted with 1-fluoro-2,4-dinitrobenzene in acetonitrile to mask the phenol (2). Finally, dioxetane formation was satisfied by reacting *in situ* generated singlet oxygen (^1^O_2_) with (2). MCH was characterized by ^1^H, ^13^C NMR and high-resolution mass spectrometry (HR-MS).

### Photophysical properties of MCH

**Fig. 2:**
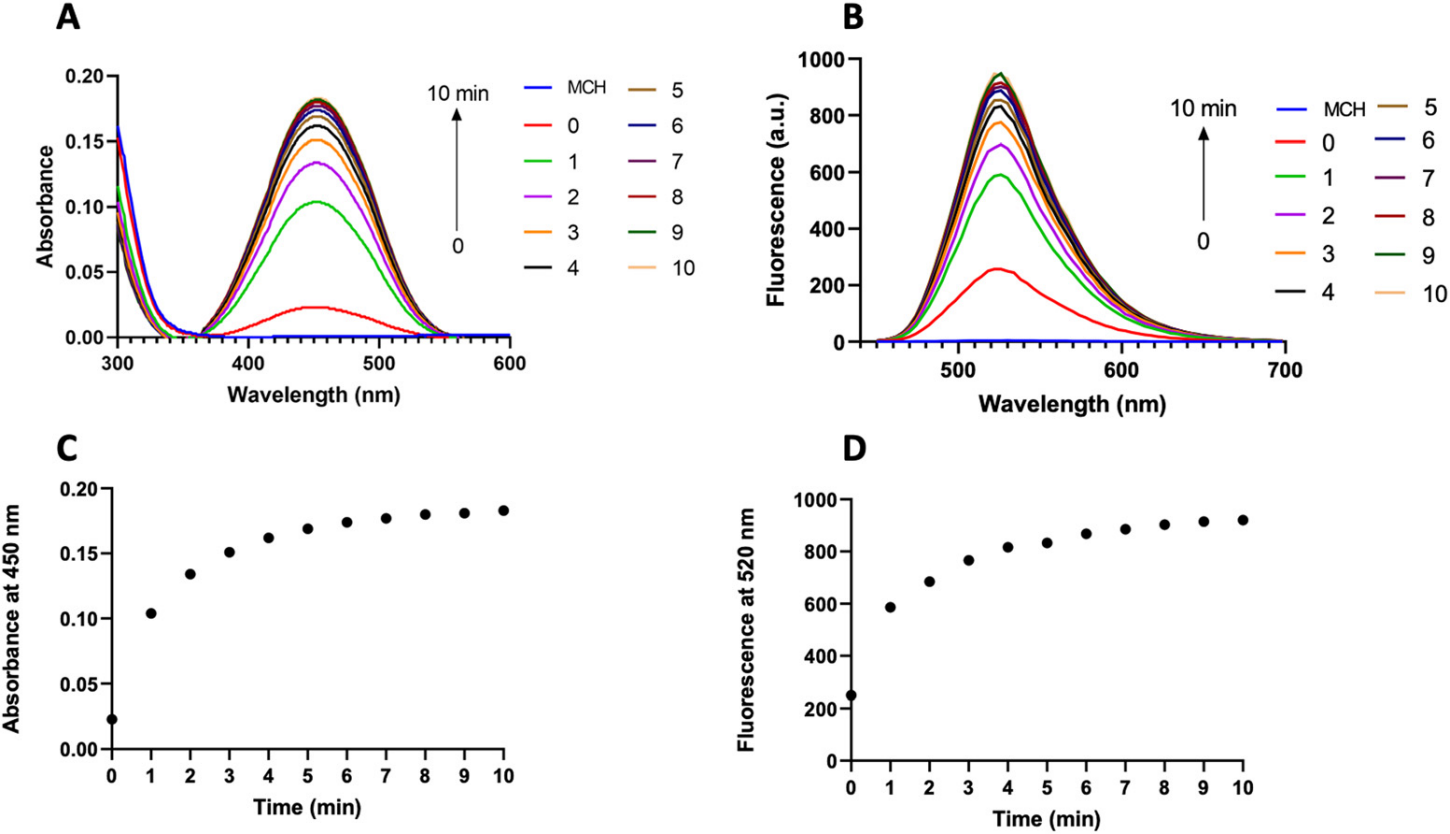
(A) Absorption and (B) fluorescence spectra of MCH (10 μM) with or without the addition of 2 eq. of H_2_S in DMSO (2% PBS, pH 7.4) and (C) corresponding absorbance at 450 nm and (D) fluorescence at 520 nm in the presence of 2 eq. of H_2_S from 0 to 10 min. *λ*_ex_ = 440 nm.

After obtaining the MCH probe, we first recorded the absorption and fluorescence spectra of MCH in DMSO (2% PBS, pH 7.4) as these are directly correlated with the chemiluminescence properties (Fig. 2).^69,87^ Upon reacting MCH (10 μM) with H_2_S (2 eq.), a time-dependent increase in both absorption (450 nm) and emission (520 nm) signals of the resulting benzoate was observed, indicating that the probe was activated with H_2_S (Fig. 2(A) and (B)). The cleavage of the masking unit was shown to be very rapid and completed in 10 minutes (Fig. 2(C) and (D)). We also synthesized the benzoate (MC-benzoate),^87^ which is expected to be released after H_2_S-induced activation, for comparison purposes. Fluorescence spectra of H_2_S-treated MCH and MC-benzoate perfectly overlapped (Fig. S1, ESI†), suggesting that the MC-benzoate was released upon activation. HR-MS analyses were also conducted to further prove the MC-benzoate release and to clarify the activation mechanism. As shown in Fig. S2 (ESI†), the signal of MCH at 874.2660 *m*/*z* disappeared and a peak at 558.1604 *m*/*z*, which belongs to benzoate (calculated as 558.1595 [M]^+^), was detected after treating MCH with H_2_S.

### Chemiluminescence characteristics of MCH

**Fig. 3:**
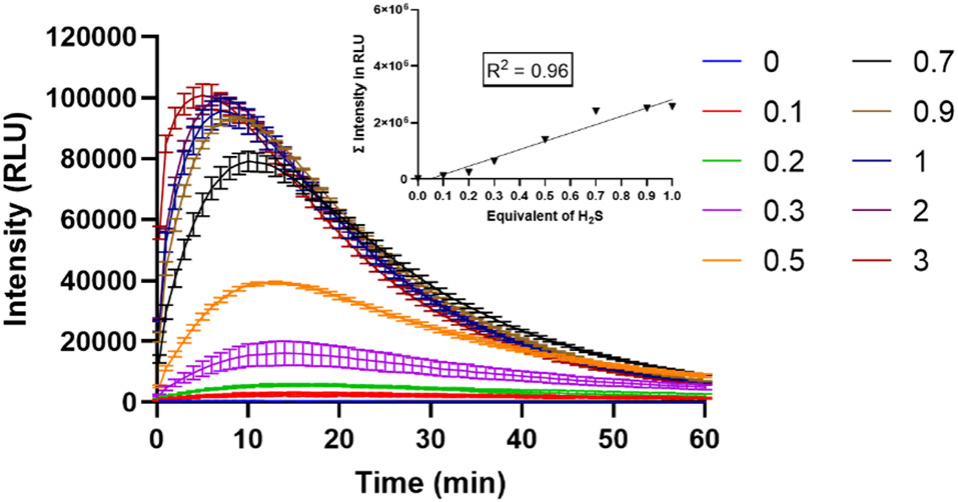
Time-dependent chemiluminescence intensity of MCH (10 μM) in the presence of various equivalents of H_2_S (0–3) (inset: plot of the total luminescence with respect to varying H_2_S concentrations in the linear range) in DMSO (2% PBS, pH 7.4).

Next, the chemiluminescence characteristics of MCH in DMSO (2% PBS, pH 7.4) were investigated. Upon treating MCH with increasing concentrations of H_2_S (0–3 eq.), a time- and concentration-dependent response was reported. The signal dramatically increased (up to 206-fold) in the first 10 minutes and then started to decrease gradually in 1 hour (Fig. 3). Chemiluminescence intensity got stronger as the H_2_S concentration increased (Fig. 3). When total luminescence signal *versus* H_2_S concentration graph was analyzed, a linear increase was shown up to 1 eq. of H_2_S (Fig. 3-inset). The detection limit of MCH was calculated to be 0.0028 μM. In the absence of H_2_S, no signal was detected, suggesting that MCH is stable in solution.

The selectivity of MCH was checked by treating it with different biologically relevant analytes, including biothiols, hydrogen peroxide (H_2_O_2_) and anions. Remarkable signal intensities were only reported in the case of H_2_S and glutathione (GSH); however, the intensity of the signal was approximately 4-fold higher in the case of H_2_S even though the GSH concentration (5 mM) was 250-fold higher than that of H_2_S (20 μM). This result clearly indicates that MCH is selective towards H_2_S (Fig. S3, ESI†).

### Detection of H_2_S in cancer cells

**Fig. 4:**
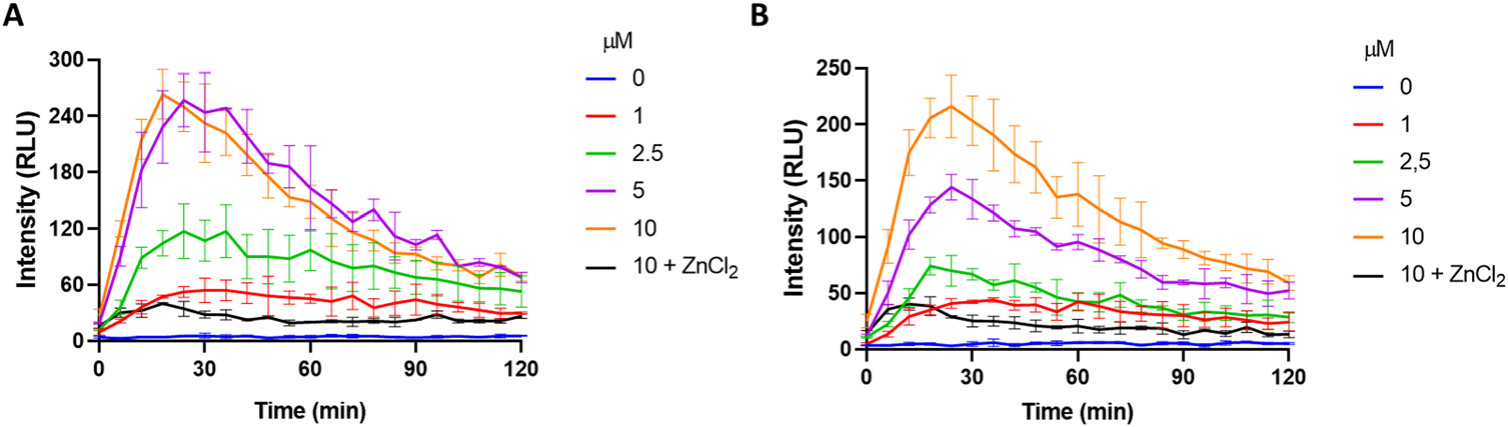
Time and dose-dependent chemiluminescence signal of MCH in (A) HCT116, (B) SH-SY5Y cells. ZnCl_2_ (300 μM) was incubated for 10 minutes before the addition of MCH. (*n* = 3).

Given the promising results obtained in solution, we next sought to investigate the performance of MCH in cell cultures. To this end, SH-SY5Y (neuroblastoma) and HCT116 (colon) cancer cells, in which the H_2_S concentration is high,^90–94^ were chosen as model cell lines. Increasing doses of MCH (0–10 μM) were incubated with the cells and time-dependent luminescence measurements were taken with a plate reader. MCH exhibited a similar kinetic profile in both cell lines. Chemiluminescence signal intensity increased in the first 30 minutes in a dose-dependent manner and then decreased slowly in 2 hours (Fig. 4), which is a typical trend for activity-based 1,2-dioxetanes. Treating cells with zinc chloride (ZnCl_2_) (300 μM), a known H_2_S quencher,^95,96^ for 10 minutes, dramatically reduced the signal intensity in both cells, indicating that H_2_S is the major endogenous trigger of the chemiluminescence (Fig. 4 and Fig. S4, ESI†). MCH was also found to be non-toxic in the working dose range as evidenced from the cell viability assay results. No sign of significant cell death was detected when both SH-SY5Y and HCT116 cells were incubated with MCH (0–100 μM) (Fig. S5, ESI†). These cumulative results show that MCH can be selectively activated in a cellular environment with endogenous H_2_S and can detect varying concentrations of H_2_S. Additionally, it is safe for bio-imaging studies.

**Fig. 5:**
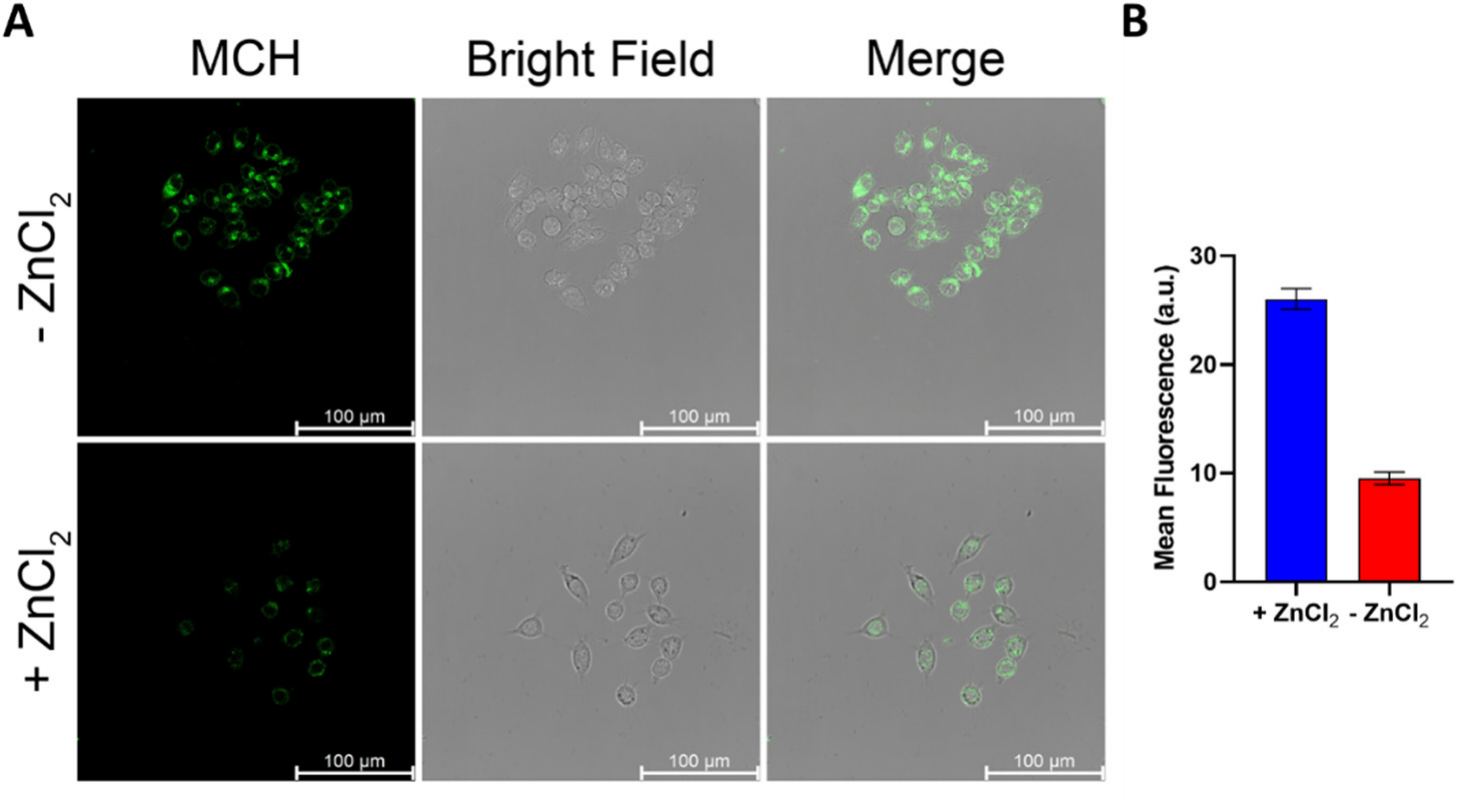
(A) Confocal microscopy images of HCT116 cells treated with or without ZnCl_2_ (300 μM) for 10 minutes. Data were collected after 30 minutes MCH (10 μM) treatment. (B) Mean fluorescence. (*n* = 4) (*λ*_ex_ = 405 nm/*λ*_em_ = 500–600 nm).

Confocal microscopy studies were performed to further evaluate the imaging potential of MCH and to investigate its subcellular localization. First, HCT116 (Fig. 5) and SH-SY5Y (Fig. S6, ESI†) cells were incubated with MCH for 1 hour, the cells were washed and then the characteristic green emission of the resulting benzoate was visualized upon irradiation of the cells with a 405 nm confocal laser. A strong fluorescence signal was detected in both cells, indicating once again that MCH can be activated with an endogenous H_2_S, and the corresponding benzoate is released. The addition of ZnCl_2_ to the cells remarkably quenched the fluorescence signal coming from the cells (Fig. 5 and Fig. S6, ESI†), which is in good agreement with the results obtained from the plate reader. Time-dependent activation of MCH was also captured under a confocal microscope with HCT116 cells. The signal appeared to be detectable at 15 min and got brighter after 30 min (Fig. S7, ESI†), which also supports the time-dependent luminescence signal data.

### Localization of MCH to mitochondria

**Fig. 6:**
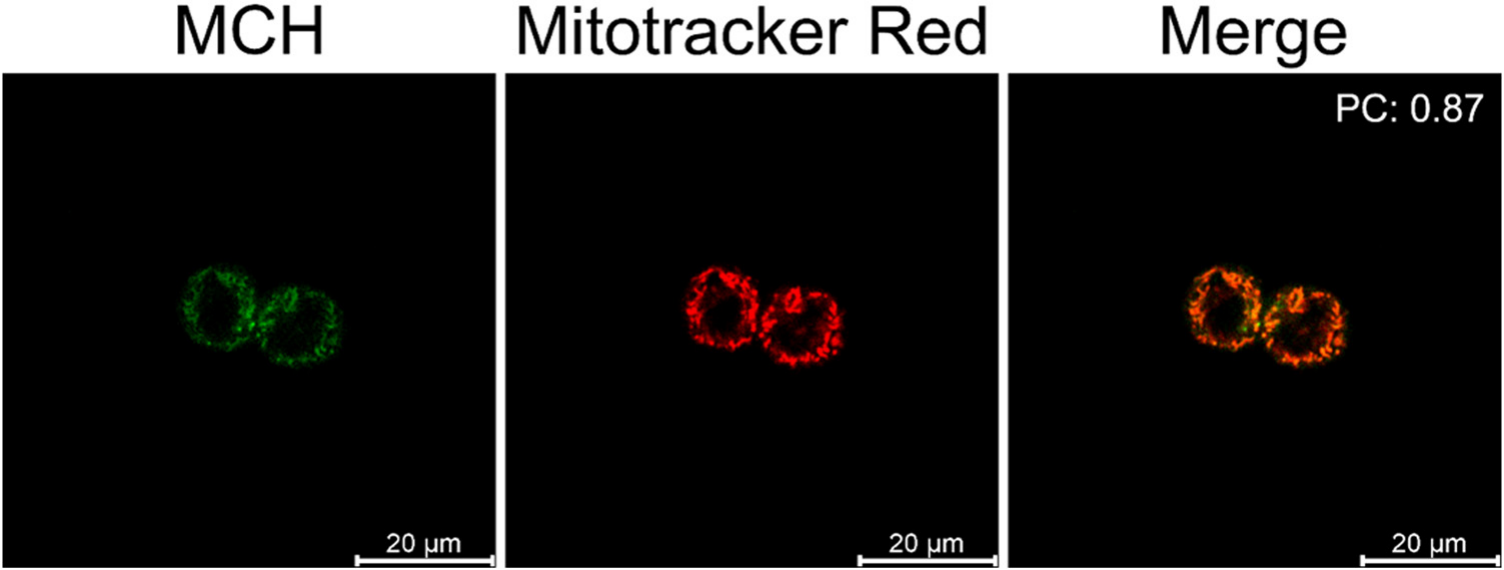
Confocal microscopy images of HCT116 cells treated with MCH (10 μM) (ex/em: 405/500–550) and Mitotracker™ Red (100 nM) (ex/em: 561/600–650). (*n* = 4)

Next, a commercially available mitochondria stain MitoTracker Red™ (100 nM) and MCH (10 μM) were co-incubated with HCT116 cells to prove their mitochondria targeting ability. As shown in Fig. 6, the emission signals coming from the red and green channels overlapped with a high Pearson's coefficient (0.87), suggesting that the probe was localized to mitochondria due to its cationic nature as expected. This also clearly supports the high mitochondrial H_2_S activity in cancer cells.

### Detection of H_2_S in human serum

**Fig. 7:**
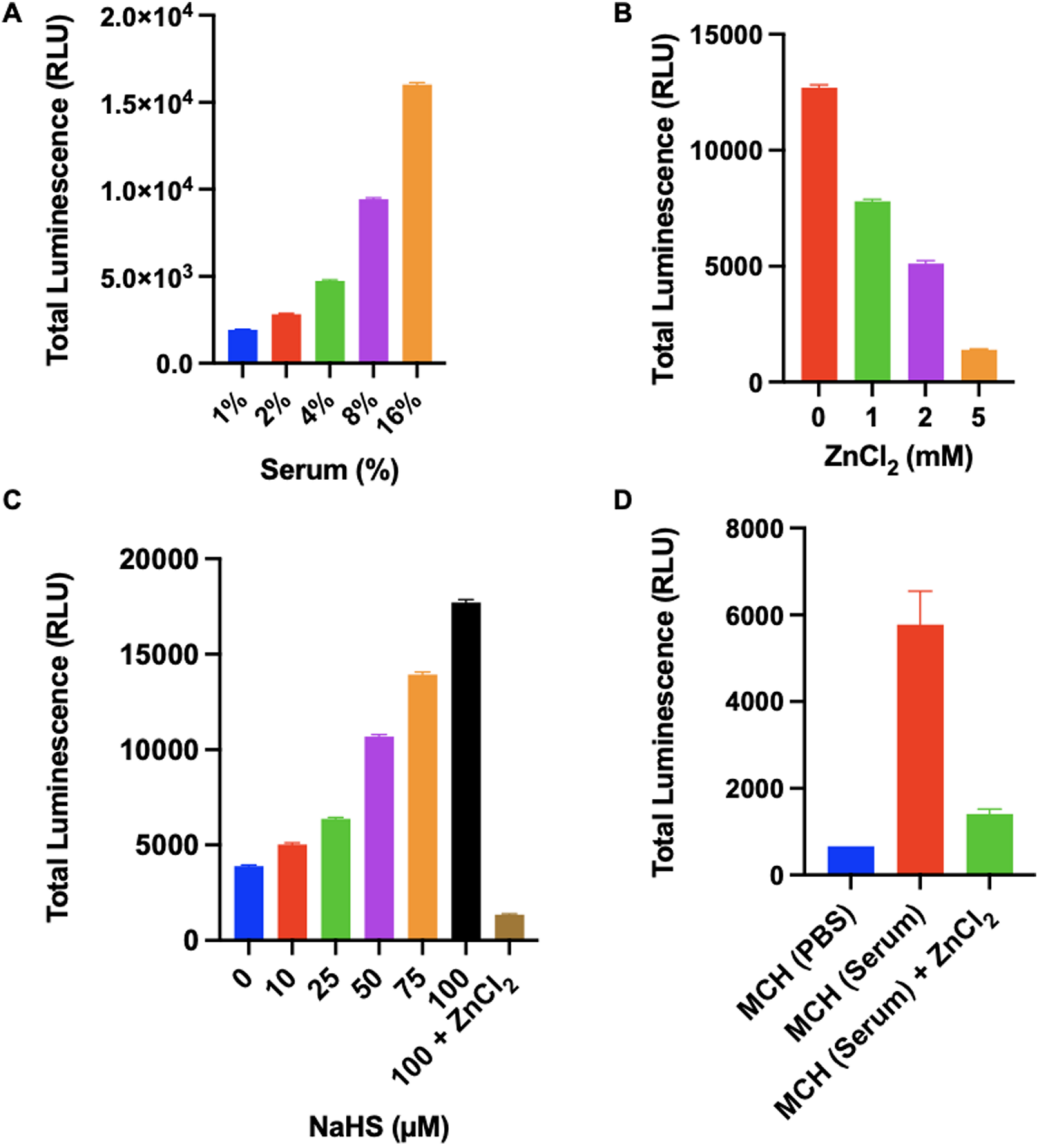
Total luminescence response of MCH (10 μM) (A) in the various (1–16%, v/v) percentages of human serum, (B) with various concentrations (1–5 mM) of ZnCl_2_ in 4% (v/v) human serum, (C) with various concentrations (10–100 μM) of NaHS in 4% (v/v) human serum in the presence and absence of ZnCl_2_ (5 mM) (D) in 10 different healthy human serum (1% DMSO, pH 7.4, *n* = 3) without and with ZnCl_2_ (5 mM). (*n* = 3)

We also tested the response of MCH in human serums, which were collected from 10 different healthy individuals (6 females, 4 males) aged between 22–39. Initially, optimization studies were performed using a serum sample. When the serum concentration in the PBS solution was increased (0–16%, v/v), a time and concentration-dependent turn-on response was reported in the chemiluminescence signal (Fig. S8, ESI†). Additionally, a linear increase was observed in the total luminescence signal as the serum concentration increased (Fig. 7(A) and Fig. S9, ESI†). To prove that the signal was raised due to H_2_S-induced activation, we treated a 4% (v/v) serum sample with increasing concentrations of ZnCl_2_. The signal decreased dramatically upon increasing the inhibitor concentration and almost complete inhibition was detected at a 5 mM ZnCl_2_ dose (Fig. 7(B)). Later, we treated the same serum sample (4%, v/v) with increasing concentrations of NaHS and checked the luminescence signal. As the NaHS concentration increased, the chemiluminescence signal was enhanced remarkably (Fig. 7(C)). Similarly, ZnCl_2_ (5 mM) treatment quenched the luminescence signal (Fig. 7(C)), proving that MCH can detect varying concentrations of H_2_S in a complex serum environment. Then, we tested MCH in 10 different serum samples to investigate its potential for extensive usage. The average signal obtained from 10 serum samples (4%, v/v) is shown in Fig. 7(D). It is worth mentioning that a comparable chemiluminescence signal was detected in all samples with slight variations (Fig. 7(D) and Fig. S10, ESI†), suggesting that MCH can function precisely in similar serum samples. Treating the samples with ZnCl_2_ (5 mM) again resulted in a strong inhibition and the total luminescence signal dropped to a level that is correlative with the signal obtained from untreated MCH in PBS solution, proving selective activation (Fig. 7(D) and Fig. S10, ESI†).

### *In vivo* tumor imaging

**Fig. 8:**
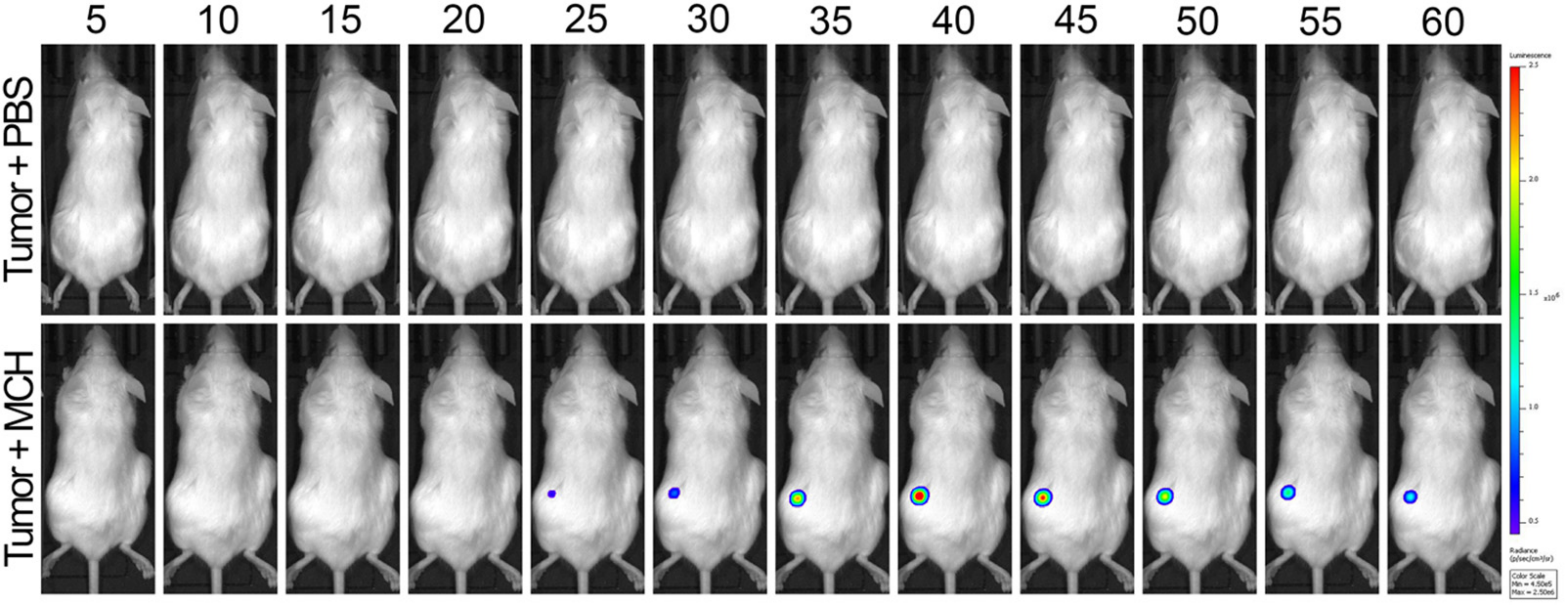
Time-dependent *in vivo* imaging. Top row: Subcutaneous injection of 100 μM 100 μL PBS as vehicle control. Bottom row: Intratumor injection of 100 μM 100 μL MCH. (*n* = 4)

Finally, MCH was utilized for *in vivo* tumor imaging. To this end, HCT116 cells were used to generate tumors on the right and left flank areas of immunocompromised mice. Later, MCH (100 μM, 100 μL) was injected intratumorally in the left flank, and time-dependent whole-body luminescence was detected under IVIS every 5 minutes for 1 hour period. The signal intensity increased during the first 40 minutes and then decreased gradually in 1 hour (Fig. 8 and Fig. S11, ESI†). As a control experiment, a group of tumor-bearing mice was injected with PBS (100 μL) subcutaneously, which did not give any signal (Fig. 8). We also checked the cytotoxicity of high concentrations of MCH in different cell lines as 100 μM of MCH was used in animal imaging. As previously mentioned, 100 μM of MCH did not cause significant cytotoxicity in HCT 116 and SH-SY5Y cancer cells (Fig. S5, ESI†). We also investigated the cytotoxicity of high concentrations of MCH in two different normal cell lines. In this direction, Vero and HGrC1 cells were incubated with various concentrations of MCH (0–100 μM). In both cells, no signs of cytotoxicity were observed, indicating that 100 μM of MCH dose is safe for bioimaging (Fig. S5, ESI†). These results support that MCH can also be utilized for *in vivo* imaging of tumor.

## Conclusions

In summary, we have introduced the first example of a H_2_S-responsive and mitochondria-targeted 1,2-dioxetane-based chemiluminescent probe (MCH), which bears a cationic TPP unit and an electron-withdrawing acrylate group. MCH exhibited a 206-fold turn-on response in its luminescence signal upon reacting with H_2_S. The probe was shown to be highly selective towards H_2_S and further utilized successfully to image endogenous H_2_S activity in SH-SY5Y and HCT116 cancer cells. MCH did not show any cytotoxicity in both cells, which is a critical parameter for a bioimaging probe. Its mitochondria-targeting capability was proved using confocal microscopy by carrying out colocalization studies with a commercially available MitoTracker dye. A strong overlap was obtained with a high Pearson coefficient. MCH also enabled us to detect H_2_S in human serums, which were collected from healthy people. Inhibition studies were performed both in cell cultures, and serum experiments to demonstrate that the source of the signal was H_2_S-triggered activation of the dioxetane. Finally, MCH was used to image tumors generated by HCT116 cells in animal models. Given the promising results obtained in *in vitro*, human serum and *in vivo* studies, we believe that mitochondria-targeted chemiluminescent probes can be further utilized to investigate the critical roles of mitochondrial analytes in various diseases especially cancer and neurodegenerative disorders. Furthermore, thanks to our design approach, the core structure can be designed easily to target different organelles. Our work along these directions is in progress.

## Experimental

### Synthesis of MCH

#### Compound 2

1^87^ (54 mg, 0.071 mmol), 1-fluoro-2,4-dinitrobenzene (41.5 mg, 0.22 mmol), and K_2_CO_3_ (77 mg, 0.55 mmol) were placed in a round bottom flask and dry ACN (10 mL) was added. The mixture was stirred at room temperature for 4 hours. After completion of the reaction, the reaction mixture was filtered, and the solvent was evaporated under vacuum. Compound 2 was obtained after column chromatography purification MeOH : DCM (5 : 95) as a pale yellow solid (41 mg, 62% yield). ^1^H NMR (500 MHz, CDCl_3_) *δ* 9.32 (t, *J* = 5.8 Hz, 1H), 8.90 (d, *J* = 2.7 Hz, 1H), 8.27 (dd, *J* = 9.3, 2.7 Hz, 1H), 7.88 (d, *J* = 8.1 Hz, 1H), 7.81–7.78 (m, 3H), 7.76–7.72 (m, 6H), 7.69–7.65 (m, 6 H), 7.62 (d, *J* = 15.7 Hz, 1H), 7.31–7.27 (m, 2H), 6.68 (d, *J* = 9.2 Hz, 1H), 3.68–3.41 (m, 4H), 3.32 (s, 3H), 3.25 (br. s, 1H), 2.06 (br. s, 1H), 2.00–1.75 (m, 14H). ^13^C NMR (126 MHz, CDCl_3_) *δ* 165.91, 155.45, 146.77, 141.71, 139.01, 138.43, 137.93, 135.41, 135.39, 133.64, 133.56, 130.81, 130.71, 129.26, 128.06, 127.44, 126.29, 122.78, 118.47, 117.78, 116.76, 57.59, 38.89, 37.11, 29.89, 29.83, 29.27, 22.51, 21.62, 21.21. MS (ES+): *m*/*z* calcd. for C_48_H_46_ClN_3_O_7_P^+^: 842.2756; HRMS found: 842.2760 [M^+^].

#### MCH

2 (30 mg, 0.032 mmol) was dissolved in DCM (30 mL) and a few milligrams of methylene blue were added to this mixture at 0 °C. During the reaction, the mixture was bubbled by oxygen and irradiated with a white LED. The reaction was monitored by RP-HPLC, upon completion the solvent was evaporated, and the product was isolated by preparative RP-HPLC (gradient of ACN in water) as an off-white solid (14 mg, 45% yield). ^1^H NMR (500 MHz, CDCl_3_) *δ* 9.72 (s, 1H), 8.91 (d, *J* = 2.7 Hz, 1H), 8.26 (dd, *J* = 9.2, 2.7 Hz, 1H), 7.94 (d, *J* = 8.4 Hz, 1H), 7.86 (d, *J* = 8.4 Hz, 1H), 7.80 (m, 3H), 7.73–7.64 (m, 12H), 7.62 (d, *J* = 15.7 Hz, 1H), 7.23 (d, *J* = 15.7 Hz, 1H), 6.67 (d, *J* = 9.2 Hz, 1H), 3.95 (s, 3H), 3.66–3.36 (m, 5H), 2.55 (br. s, 1H), 2.10–1.87 (m, 14H). ^13^C NMR (126 MHz, CDCl_3_) *δ* 165.69, 154.96, 147.35, 141.94, 135.49, 135.47, 134.32, 133.47, 133.39, 132.25, 130.79, 130.69, 130.48, 129.82, 129.29, 128.68, 127.95, 126.17, 122.79, 118.34, 117.65, 116.77, 52.98, 47.11, 39.39, 36.43, 29,88, 29.84, 29.81, 27.57, 22.84, 22.43. MS (ES+): *m*/*z* calcd for C_48_H_46_ClN_3_O_9_P^+^: 874.2655; HRMS found: 874.2660 [M^+^].

### Cell culture experiments

The HCT116, SH-SY5Y, Vero and HGrC1 cells were grown in a high glucose DMEM supplemented with 2% penicillin–streptomycin and 10% FBS. The cells were passaged with DPBS and Trypsin-EDTA at 70% confluency and incubated in an Eppendorf Galaxy 170S incubator at 37 °C and 5% CO_2_.

For the kinetic measurements, HCT116 and SH-SY5Y cells were seeded in dark-sided, clear-bottomed 96-well plates (10 000 cells per well) and incubated overnight. One of the groups was treated with ZnCl_2_ (300 μM) for 10 minutes and washed with PBS. Then, the cells were treated with MCH (0–10 μM) in PBS (1% DMSO, v/v, pH 7.4). The inhibition group was treated with MCH (10 μM) in PBS (1% DMSO, v/v, pH 7.4). The kinetic luminescence measurement was started immediately after the treatment and the chemiluminescence signal was detected using a Biotek Synergy H1 MF microplate reader. (*n* = 3, technical replicates).

HCT116 cells were seeded into 35 mm glass bottom confocal plates (10 000 cell per plate) and incubated overnight. One of the groups was treated with ZnCl_2_ (300 μM) for 10 minutes and washed with PBS. Following the inhibition period, the cells were treated with MCH (10 μM) and washed with PBS, after half an hour. Time-dependent confocal images of HCT116 cells were captured at fixed-interval *z* stack before and after the addition of MCH, and recorded for 30 minutes. For the colocalization study, cells were treated with MCH (10 μM) for half an hour and washed with PBS. Then, the cells were stained with MitoTracker Red (100 nM) for 15 minutes and washed with PBS. Finally, confocal images were collected using a Leica DMI8 SP8 Inverted Confocal Microscope. Colocalization was analyzed using Image J software. (*n* = 4, technical replicates).

To perform the cell viability assay, cells at confluency were seeded into a dark-sided clear-bottomed 96-well plate at a density of 1 × 10^4^ cells per well and allowed to incubate overnight. The following day, the cells were treated with various concentrations of MCH in DMEM (1% DMSO, v/v). After an hour, the MCH-containing medium was replaced with fresh DMEM, and the cells were further incubated for 23 hours. To measure cell viability, 44 μL of fresh CellTiter-Glo reagent was added to each well. (*n* = 3, technical replicates).

### Monitoring H_2_S level in human serum

The serum experiments were performed with the approval of the local ethical committee of Koç University. Informed consent was obtained from all participants and experiments were performed according to the guidelines of the Committee on human research at Koç University, Türkiye. 10 different healthy participant serum samples have been obtained from a previous study, where methodological details were reported in detail.^97^ For optimization studies, serums were diluted with PBS in different concentrations (1–16%), then 10 μM MCH was added to each well. Time-dependent luminescence response was monitored for 2 hours using a Biotek Synergy H1 MF microplate reader. Later, 10 human serum samples were diluted with PBS to a final 4% concentration. For those treated with ZnCl_2_, 5 mM ZnCl_2_ was added. All samples were incubated at 37 °C for 30 min, then 10 μM MCH was added to each well. Subsequently, time-dependent luminescence response was monitored 2 hours using a Biotek Synergy H1 MF microplate reader. (1% DMSO, pH 7.4, *n* = 3, Fig. 7(A)–(C): technical replicates, Fig. 7(D): independent replicates).

### *In vivo* imaging

The institutional ethical committee of Koç University approved all *in vivo* experiments. All animal experiments were performed according to the guidelines of the Animal Research Local Ethics Committee at Koç University, Türkiye. For these experiments, 6-8-week-old non-obese diabetic/severe combined immunodeficiency (NOD/SCID) mice were anesthetized using isoflurane. Tumor formation was induced subcutaneously for the administration of HCT116 cells to the mice. Each injection consisted of 2.5 × 10^6^ cells in a 100 μL volume of PBS, and injections were made into the right and left flank area using a 23-gauge needle. Once the tumor diameter reached approximately 0.3–0.5 cm, 100 μL of 100 μM MCH was injected intratumorally. In control experiments, healthy mice were injected 100 μL of 100 μM MCH subcutaneously. For the control experiments with tumorigenic mice, 100 μL of 100 μM PBS was injected as a vehicle control intratumorally into another group of tumor-bearing mice. The kinetic luminescence measurement was initiated immediately following the injections, and *in vivo* imaging was performed using the PerkinElmer IVIS Lumina Series III instrument. (*n* = 4, technical replicates).

## Conflicts of interest

There are no conflicts to declare.

## Supplementary Material

CB-004-D3CB00070B-s001
