# Supplementary material for: A mitochondria-targeted chemiluminescent probe for detection of hydrogen sulfide in cancer cells, human serum and in vivo
Source: RSC Chem Biol. 2023 Jul 18;4(9):675–84. doi: 10.1039/d3cb00070b (PMC10467614; doi:10.1039/d3cb00070b)
Supplement: CB-004-D3CB00070B-s001 [file CB-004-D3CB00070B-s001.pdf]

Electronic Supplementary Material (ESI) for RSC Chemical Biology.
This journal is © The Royal Society of Chemistry 2023

Supporting Information

A mitochondria-targeted chemiluminescent probe for detection of hydrogen sulfide in cancer cells, human serum and *in vivo*

Hande Gunduz,[ab1] Toghrul Almammadov,[b1] Musa Dirak,[b1] Alperen Acari,[c1] Berkan Bozkurt[cd] and Safacan Kolemen*[bce]

[a]Nanofabrication and Nanocharacterization Centre for Scientific and Technological Advanced Research, Koç University, Istanbul 34450 Türkiye
[b]Department of Chemistry, Koç University, Rumelifeneri Yolu, Istanbul 34450, Türkiye
[c]Koç University Research Center for Translational Medicine (KUTTAM), Istanbul 34450, Türkiye
[d]Graduate School of Health Sciences, Koç University, Rumelifeneri Yolu, Istanbul 34450, Türkiye
[e]Koç University Surface Science and Technology Center (KUYTAM), Istanbul 34450, Türkiye
[1]These authors contributed equally.

E-mail: skolemen@ku.edu.tr

1. Materials and Instruments

All reagents were commercially available and used without further purification unless otherwise noted. All dry solvents were obtained by using standard procedures and dry THF was obtained from Inert PureSolv solvent drying system. Reactions needed to be done under inert atmosphere were done in a Schlenk line and Argon was used as an inert gas. For Thin layer chromatography (TLC Merck Silica Gel 60 F254) commercially prepared 0.25 mm silica gel plates were used, and visualization of the compounds were done by UV light. Silica gel 60 (Merck 230-400 mesh) was used for column chromatography experiments. The relative proportions of solvents in chromatography solvent mixtures refer to the volume:volume ratio. The reaction monitoring and purification of the target probe were accomplished by reverse-phase high pressure liquid chromatography (RP-HPLC): C18 4μm, 150x4.6mm, eluent given in parentheses; preparative RP-HPLC: C18 5 μm, 250x21.2 mm, eluent given in parentheses. ^{1}H-NMR and ^{13}C-NMR analyses were recorded by a 500 MHz Bruker Ascend magnet equipped with an Avance NEO console spectrometer using $CDCl_3$ as the solvent. The chemical shifts are reported in parts per million (ppm) downfield from an internal TMS (trimethylsilane) reference. Coupling constants (J) are reported in hertz (Hz), and the spin multiplicities were specified by the following symbols: s (singlet), d (doublet), t (triplet), and m (multiplet). NMR spectras were processed with MestReNova program. Mass spectra were recorded on Waters Vion QTOF mass spectrometer. HPLC analyses were performed on an Agilent 1260 series. UV-Vis and fluorescence spectrums were acquired on Shimadzu UV-Vis-NIR spectrophotometer and Agilent Cary Eclipse spectrophotometer, respectively. Chemiluminescence signal was detected by Biotek Synergy H1 MF microplate reader. Confocal images were taken by using Leica DMI8 SP8 Inverted Confocal Microscope. Perkin Elmer IVIS Lumina Series III was used for *in vivo* imaging studies.

2. Photophysical Characterization

Photophysical Characterization Details:

Absorbance and fluorescence spectra of the **MCH** (10 μM), before and after treatment with H_2S (20 μM) in DMSO (2% PBS, pH 7.4), were recorded every minute during a total 10-minute time span (λ_{ex} = 440 nm). Fluorescence spectra of MC-benzoate were captured with similar conditions and superimposed with the spectra of **MCH** (10 μM) treated with H_2S (20 μM).

Chemiluminescence Signal:

Chemiluminescence signal of **MCH** (10 μM) was measured in the absence or presence of varying concentrations of H_2S (1-30 μM) in DMSO (2% PBS, pH 7.4). Chemiluminescence response of **MCH** was captured every minute for a total of 60 minutes and no filters were used.

Selectivity:

Chemiluminescence response of the **MCH** (10 μM) against other biologically relevant or competing analytes was measured to prove its selectivity towards H_2S. **MCH** (10 μM) treated with GSH (5 mM), L-Cys (5 mM), H_2O_2 (1 mM), $Na_2S_2O_3$ (1 mM), SO_4^{2-} (1 mM), KSCN (1 mM), NaF (1 mM), OAc^- (1 mM) and $NaNO_2$ (1 mM) and the peak luminescence signal of each analyte was recorded.

Limit of Detection:

The limit of detection (LOD) was calculated by the following formula:

$$LOD = \frac{3 * \sigma}{m}$$

where σ stands for the standard deviation of the total chemiluminescence intensity of seven separate replicates of **MCH** (10 µM), and m refers to the slope of the total chemiluminescence signal versus the concentration of H_2S (0-1 eq.).

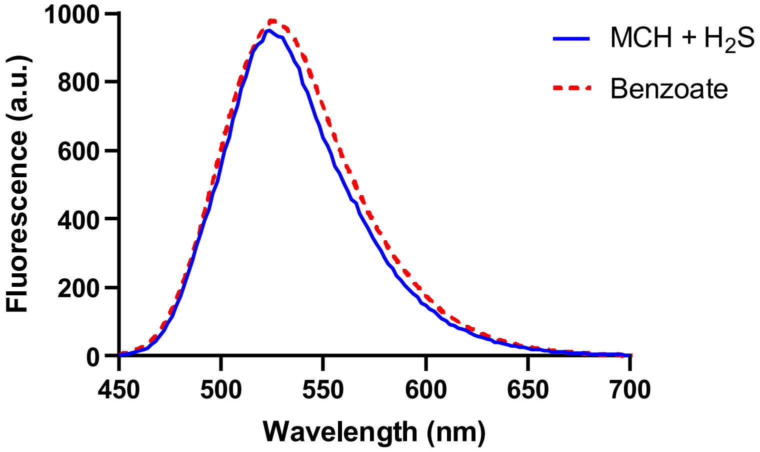

Figure S1. Fluorescence spectra of H_2S-treated (2 equivalents) **MCH** and **MC-benzoate** ester in DMSO (2% PBS, pH 7.4). λ_{ex} = 440 nm.

3. HR-MS Analyses and Activation Mechanism

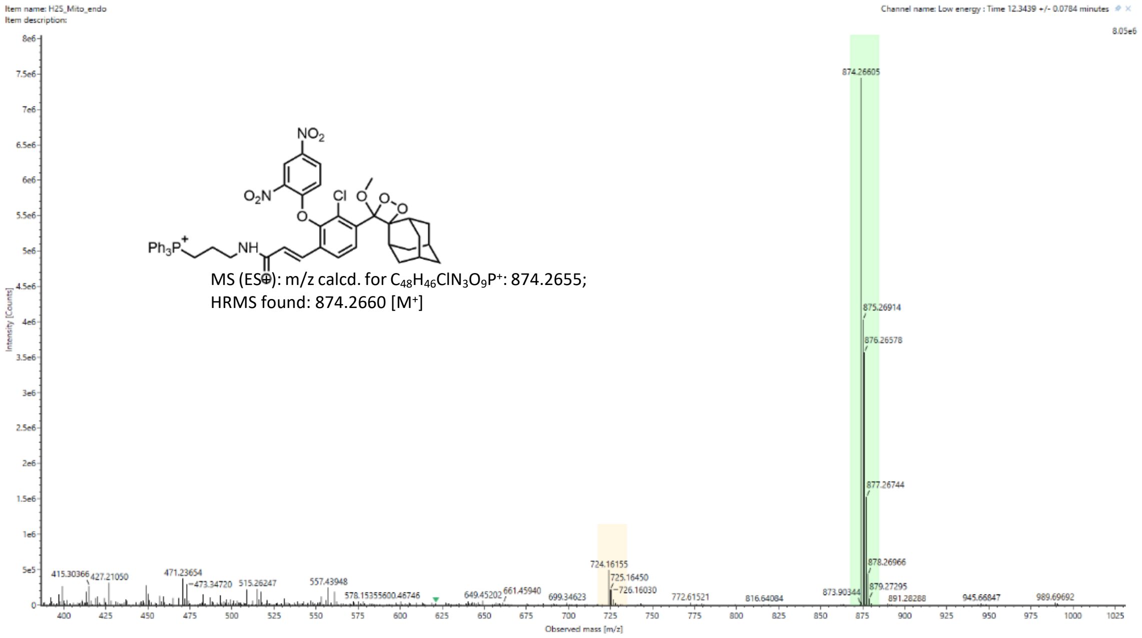

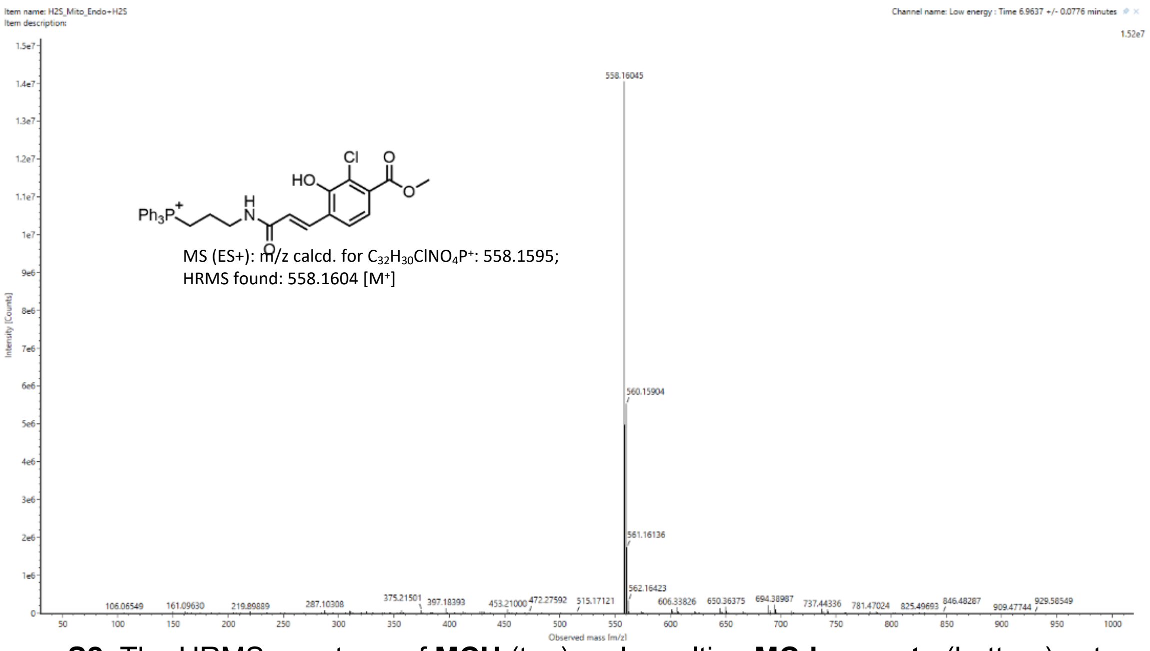

Figure S2. The HRMS spectrum of **MCH** (top) and resulting **MC-benzoate** (bottom) ester after H_2S treatment.

4. Selectivity

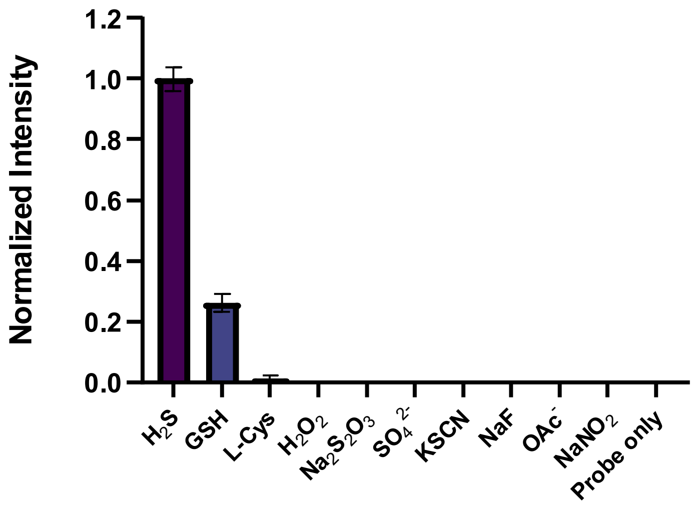

Figure S3. Luminescence intensity of **MCH** (10 μM) with various analytes. (n=3)

5. Detection of the Chemiluminescence Signal

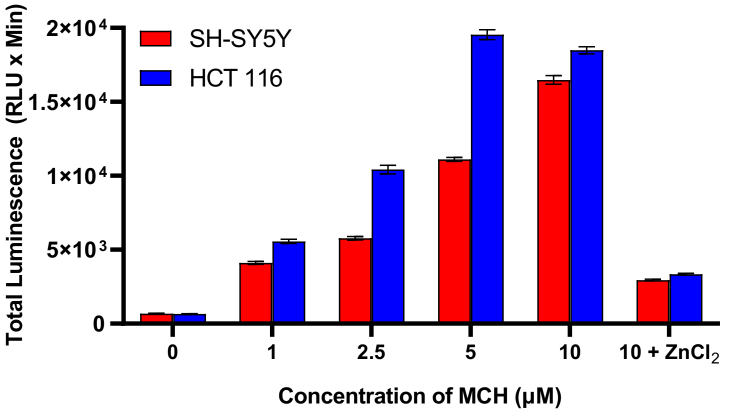

Figure S4. Total luminescent signal of **MCH** in HCT116 and SH-SY5Y (2 h). (n=3)

6. Cell Culture Studies

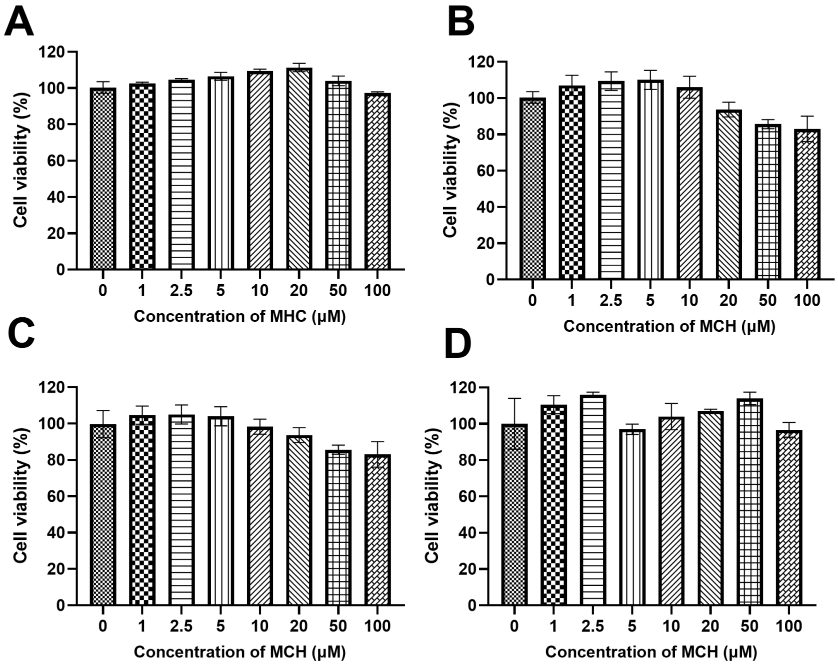

Figure S5. Cell viability of A) Vero, B) HCT116, C) SH-SY5Y and D) HGrC1 cells treated with various concentration of **MCH** (0–100 µM). (n=3)

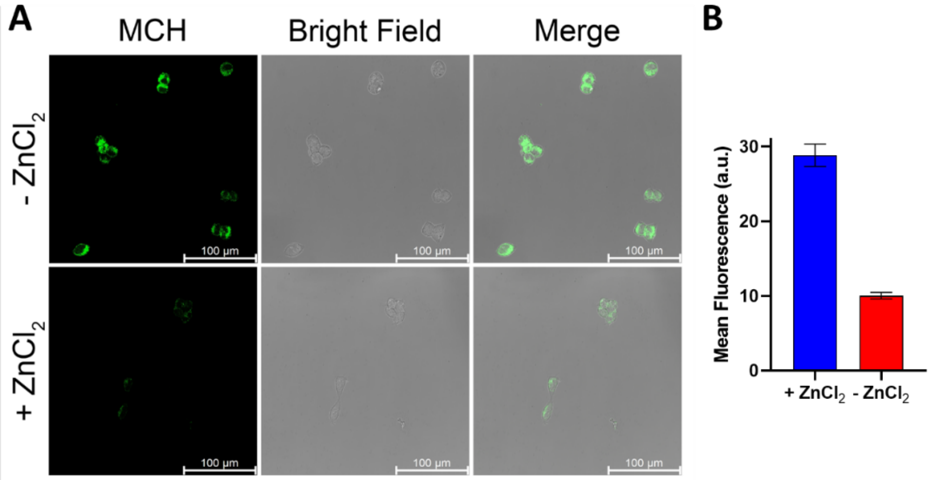

Figure S6. A) Confocal microscopy images of SH-SY5Y cells treated with and without $ZnCl_2$ (300 μM) for 10 minutes. Data was collected after 30 minutes **MCH** (10 μM) treatment. **B)** Mean fluorescence. (n=4) (λ_{ex}= 405 nm / λ_{em}= 500-600 nm). (n=3)

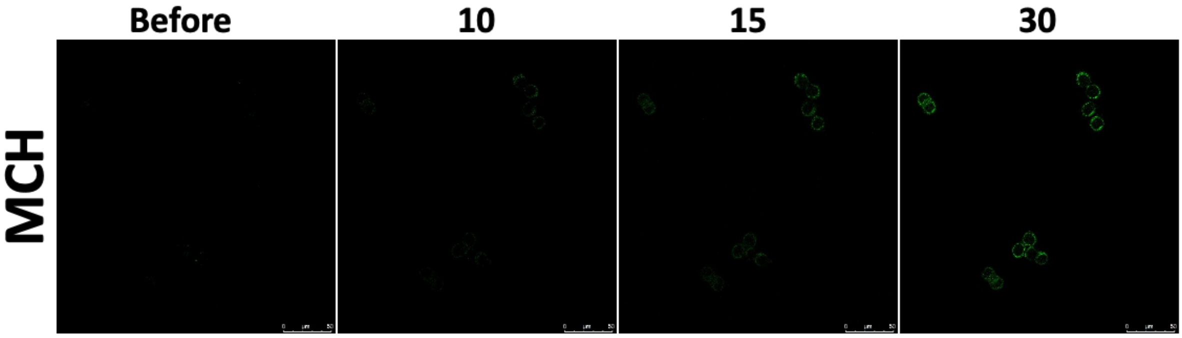

Figure S7. Confocal images of **MCH** before and after treatment of HCT116 cells captured over the course of 30 minutes. Scale bar: 50 μM.

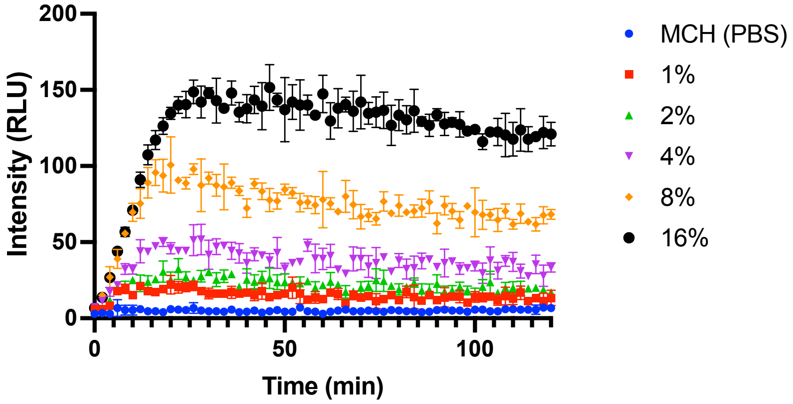

Figure S8. Time dependent luminescence response of **MCH** (10 μM) in the various (1%-16%) percentages of human serum in PBS (1% DMSO, pH 7.4, n=3).

7. Serum Experiments

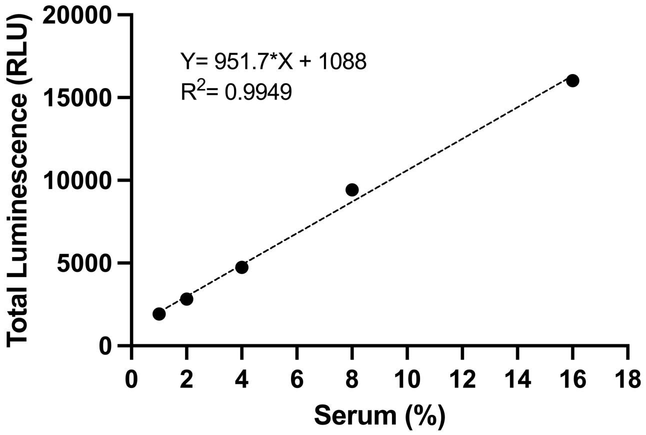

Figure S9. Calibration curve of **MCH** (10 μM) in the various (1%-16%) percentage of human serum. (n=3)

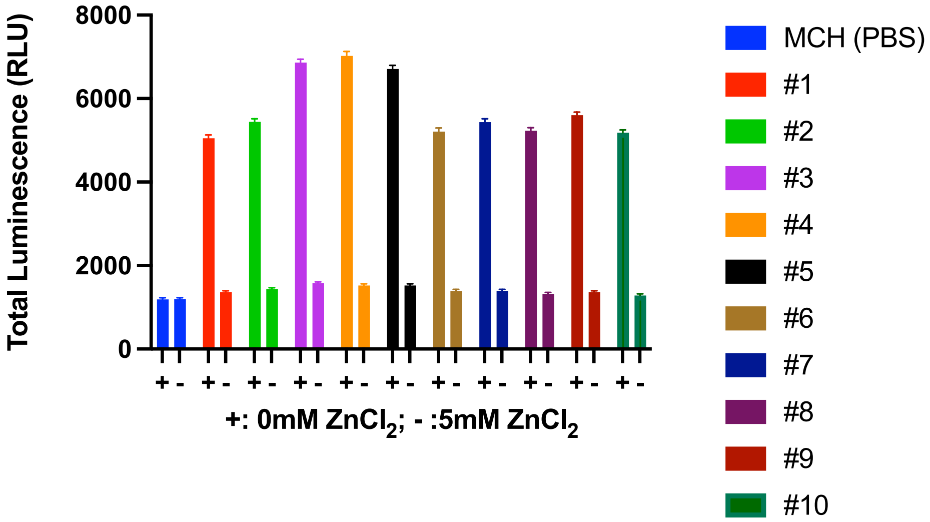

Figure S10. Total luminescence response of **MCH** (10 μM) in 10 different human serum (%4). (n=3)

8. *In vivo* Imaging

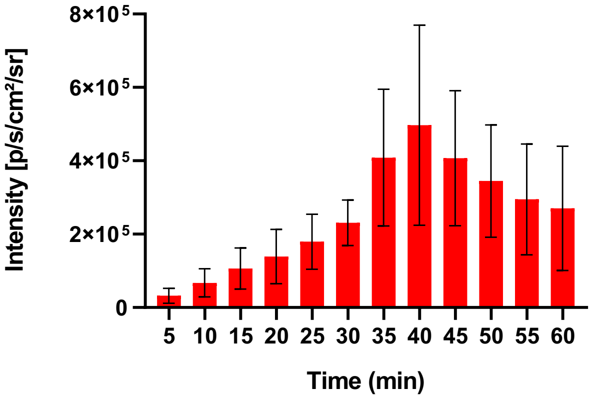

Figure S11. Time dependent luminescence intensity in the tumor region. (n=4)

9. NMR and HR-MS Spectra

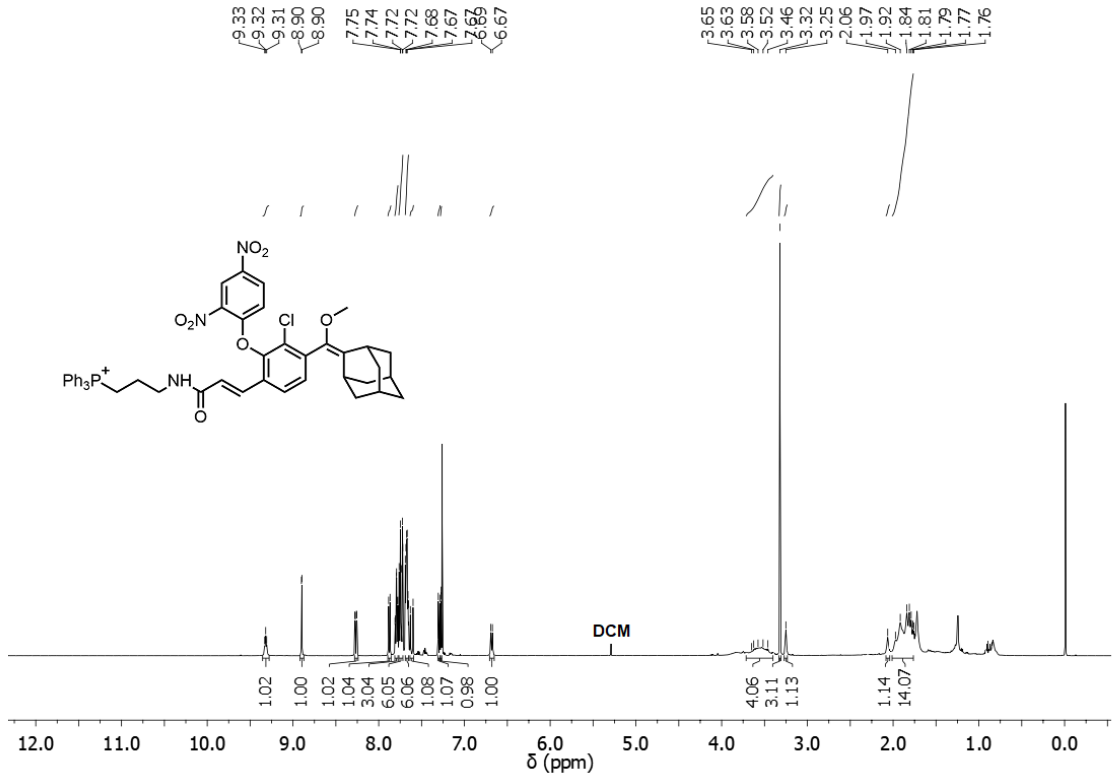

Figure S12. ^{1}H NMR spectrum of compound **2** in $CDCl_3$.

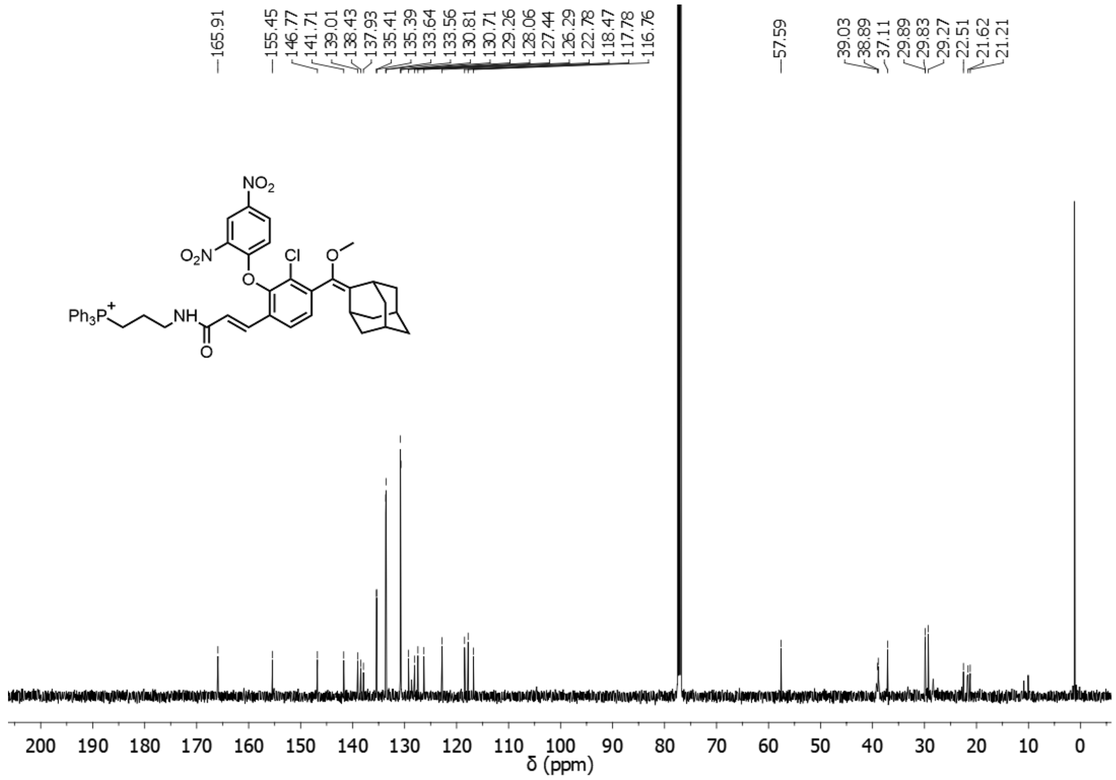

Figure S13. ^{13}C NMR spectrum of compound **2** in $CDCl_3$.

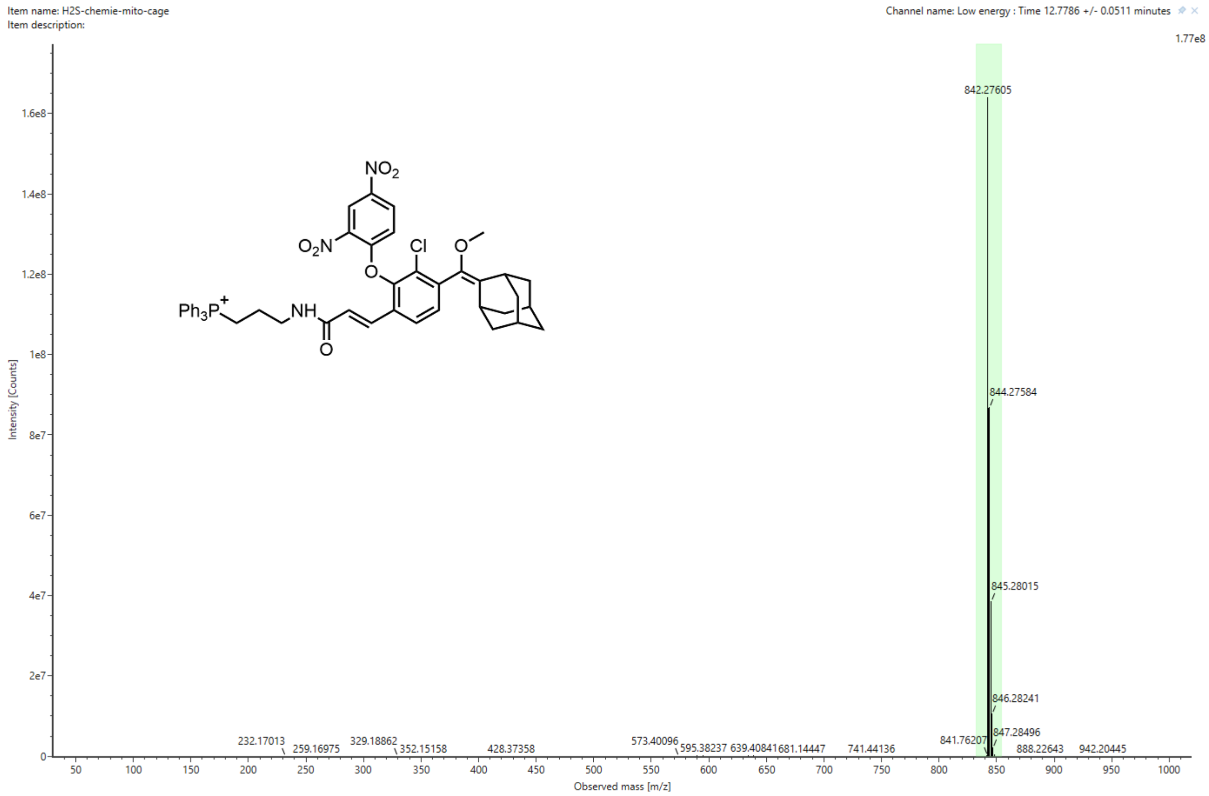

Figure S14. HRMS spectrum of compound **2**.

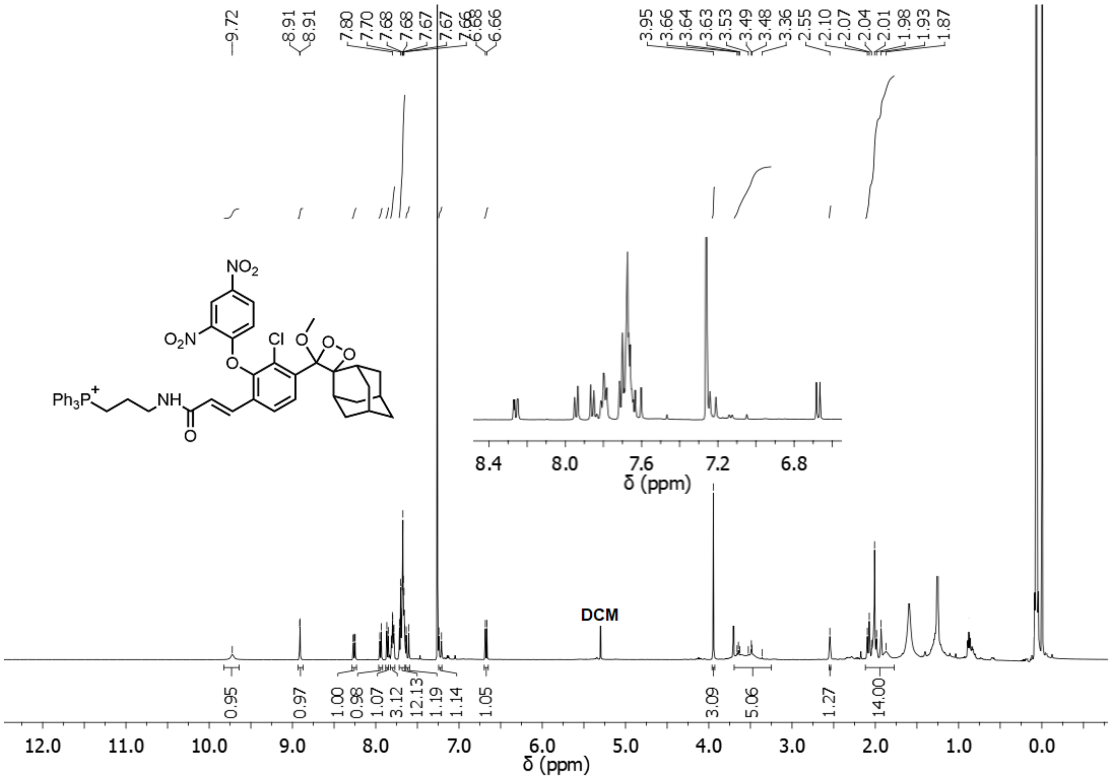

Figure S15. ^{1}H NMR spectrum of compound **MCH** in $CDCl_3$.

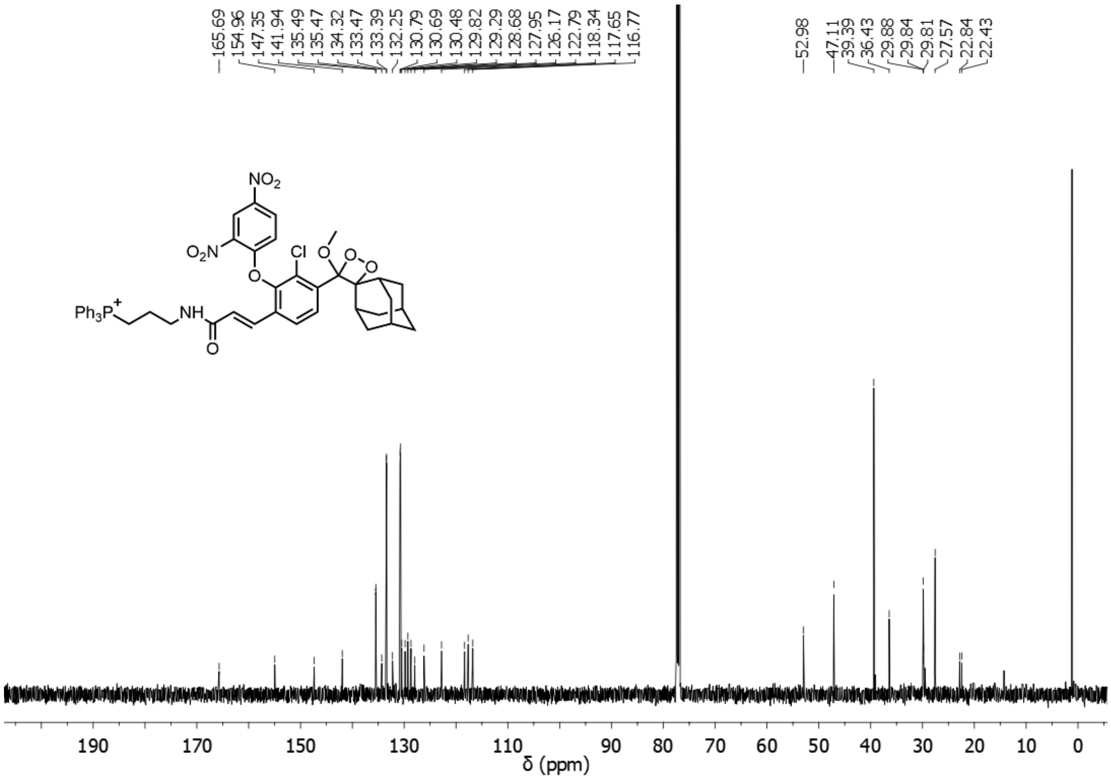

Figure S16. ^{13}C NMR spectrum of compound **MCH** in $CDCl_3$.
